# Microreactors—A Powerful Tool to Synthesize Peroxycarboxylic Esters

**DOI:** 10.3390/molecules21010005

**Published:** 2015-12-22

**Authors:** Tobias Illg, Annett Knorr, Lutz Fritzsche

**Affiliations:** 1 Fraunhofer ICT-IMM, Carl-Zeiss-Straße 18-20, 55129 Mainz, Germany; 2 Federal Institute for Materials Research and Testing (BAM), Unter den Eichen 87, 12205 Berlin, Germany; annett.knorr@bam.de (A.K.); lutz.fritzsche@bam.de (L.F.)

**Keywords:** microreactor, peroxycarboxylic ester, multiphase, peroxide, continuous processing, process safety, flow chemistry

## Abstract

The synthesis of peroxycarboxylic esters, as one subgroup of organic peroxides, is characterized by a high thermal hazard potential regarding process safety. In case of failure in the production process, e.g., if the heat of reaction cannot be removed sufficiently fast, decomposition reactions can be triggered, and as a result, remarkable amounts of heat and gas can be released and can cause a high extent of damage. Multifarious technical and organizational measures are necessary to ensure the safe industrial production of peroxides. With the introduction of microreaction technology plenty of possibilities have been opened to carry out highly exothermic reactions in smaller volumes and with more efficient heat removal. In this paper we report the application of three different microstructured reactors, representing different mixing strategies, to synthesize two peroxymonocarboxylic esters, namely *tert*-butyl peroxypivalate and *tert*-butyl peroxy-2-ethylhexanoate. The following reactor types were considered: an orifice microreactor, a split and recombine microreactor and a capillary tube reactor in combination with ultrasonication. The efficiency of the two phase liquid/liquid reaction is expressed in comparison of conversion and selectivity. With microreaction technology a remarkable increase in space-time-yield, ranging from 12,500 kg·m^−3^·h^−1^ to 414,000 kg·m^−3^·h^−1^, is achieved.

## 1. Introduction

Since the 1950s, organic peroxides have been intensively used in the chemical industry, especially as initiators in the production of polymers [1]. This class of substances easily decomposes to free radicals and therefore, is widely applied in free radical polymerizations, as cross linking and bleaching agents [2]. In general, all organic peroxides can be considered as derivatives of hydrogen peroxide in which one or both hydrogen atoms are replaced by organic groups. The structure of these groups controls the reactivity of the organic peroxide. This allows tailoring the structure (and hence reactivity) to suit the needs of a specific application. Approximately 100 different organic peroxides are commercially produced and are available in more than 300 different formulations [3]. A common practice for the comparison of organic peroxides, concerning their thermal activity, is the specification of their half-lifetime, which is the time required to reduce the original content by 50% at a given temperature. The corresponding half-life temperatures for e.g., 10 h range from 20 °C to 200 °C approximately. The oxygen-oxygen bond is weak and the bond enthalpy Δ*H* is in the range of about −84 kJ·mol^−1^ to −184 kJ·mol^−1^ [3]. Cleavage of the peroxide bond can be induced, e.g., by light, heat, mechanical stress and reactive contaminants. For pure peroxides the decomposition process can be mostly described by first order reaction kinetics. The resulting decomposition is normally accompanied by a large release of energy and gas, and is hereby associated with a large extent and severity of damage [4,5,6]. In the production of peroxides thus not only the heat generation by the synthesis but also the potential start of decomposition contribute to the hazard potential of the process. In this paper we focus on the microreactor-assisted continuous synthesis of *tert*-butyl peroxypivalate (TBPP) and of *tert*-butyl peroxy-2-ethylhexanoate (TBPEH) as two representatives of the large group of esters of peroxymonocarboxylic acids. They are used as resin hardeners and as initiators for polymerisation reactions [7,8,9].

### 1.1. A Short Survey of the History of the Perester Synthesis

Until 1946, only six peresters were known: ethyl peracetate, dimethyl-, diethyl-, and diisopropylperterephthalate, *S*-methyl perester-*t*-methyl campherate and *trans*-9-decalyl perbenzoate. The first five have been prepared from the barium salt of the hydroperoxide and the corresponding acid chloride, while the last one has been prepared in pyridine from the hydroperoxide and benzoyl chloride [10]. The most important and industrially most frequently employed method for the synthesis of peroxycarboxylic esters is the reaction of a carboxylic acid halide with a hydroperoxide [2]. This method was primarily described in 1946 and enabled the synthesis of eight different tertiary butyl esters that could not be produced before. This synthetic route paved the way for a variety of industrial processes for the production of organic peresters. During the last years different processes have been patented. Most of them use the same route or some slight modification of the one developed by Milas and Surgenor in 1946 [10].

### 1.2. Microreactors—A Powerful Tool for Organic Peroxide Production

How do hazardous processes benefit from micro-process technology? Beneficial for these processes, amongst others, is the small reaction volume of microreactors resulting in a small hold-up of reactive chemicals. Furthermore, the superb temperature control as a consequence of the very large surface-to-volume ratio which is in the range of 10,000 m^2^·m^−3^ to 50,000 m^2^·m^−3^ [11] decreases the risk of a thermal runaway reaction. Additionally, the possibility of on-demand and/or on-site production of hazardous substances helps to increase the process reliability, because transportation and storage of hazardous substances can be eliminated. The use of micro-process technology has become more and more common beyond the realm of the academic world. For example, its use within the concepts of decentralized, mobile and flexible manufacturing plants is addressed in several publicly funded projects like CoPIRIDE [12], F^3^ Factory [13], and POLYCAT [14]. The use of a container-like infrastructure in combination with e.g., microreactor technology to do process development has been strongly pushed into the market in the last years, e.g., by companies like Evonik Industries [15] and Bayer Technology Services [16].

Industry has already started to use microstructured equipment to produce organic peroxides. In 2007, the synthesis of a variety of organic peroxides, supported by microstructured equipment, was described [17]. The main driver for this invention was to increase the process safety by a reduction of reaction volume. The concept of on-demand and/or on-site production was picked up for the continuous synthesis of unstable performic and peracetic acids in [18,19]. The considered concept is a plate type reactor, and the presented design study would have a capacity of 100 t·a^−1^ of performic acid and 170 t·a^−1^ of peracetic acid. In [20,21] the continuous peroxidation of ethyl methyl ketone was done in a specially designed T-mixer-loop set-up. Under optimized conditions, the reaction is completed in seconds and meets the standard for industrial applications. The improved temperature control in this type of set-up is a key feature to obtain good yields. The microreactor-assisted synthesis of TBPP using a simple capillary reactor equipped with orifice type inserts is described in [22]. This concept has been developed further and is described in reference [23] and in much more detail in reference [24] as an orifice microreactor. Here, specially designed orifices located at certain positions in the reaction channel are used for re-emulsification. In reference [25] the transformation of a semi-batch perester synthesis, that of TBPEH, into a continuous process using a capillary reactor in combination with ultrasonication is described.

This short review on the applicability of microreactors and their benefits for hazardous reactions illustrates that this technology has already started to alter the chemical world beyond the chemical laboratory. The processing of organic peroxides is accompanied by different challenges and by a number of safety issues that have to be taken into account. That the high hazardous potential of these substances should not be underestimated is illustrated by an accident that occurred in 2003 at the Catalyst System Inc. site in Ohio [26]. In this case, a benzoyl peroxide explosion occurred and caused significant damage to buildings; luckily only one person was injured. The German Commission on Process Safety has released a technical rule (TRAS 410) for the identification and control of exothermic chemical reactions [27]. The main focus of this guideline is on the potential hazards caused by a chemical reaction due to heat generation/release, volume of gas generation/release and nature plus quantity of involved substances. In reference [28] this technical guide was applied to access the thermal process safety of the synthesis of TBPP done in semi-batch mode. As one result it was found that the process temperature and the temperature at which the organic perester starts to decompose lay very close together. Hence, only small deviations of process conditions can be allowed. For safe processing a lot of process monitoring and control technology is needed. In this work it was recommended to transform the process into a continuous one supported by micro process technology to achieve process safety benefits.

## 2. Results and Discussion

### 2.1. Peroxycarboxylic Ester Synthesis Using Different Types of Microreactors and Concepts for Emulsification

**Scheme 1:**
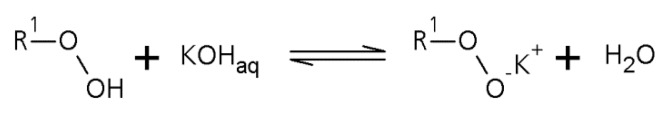
First step of the perester synthesis: Deprotonation of the hydroperoxide with potassiumhydroxide to the corresponding potassium peroxide.

**Scheme 2:**
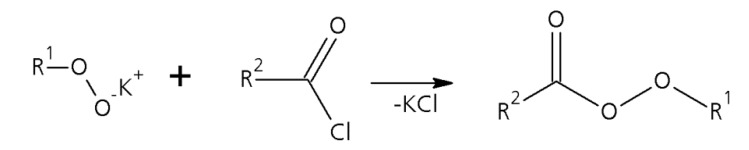
Second step of the perester synthesis: Formation of the perester by reaction between the potassium peroxide with the acid halide.

In this paper we focus on the microreactor-assisted continuous synthesis of TBPP and of TBPEH as two representatives of the large group of esters of peroxymonocarboxylic acids. These reactions can be divided into two separate steps, first the deprotonation of the hydroperoxide with potassium hydroxide (single phase reaction) and second the conversion of the formed organic potassium salt with an acid halide to form the corresponding perester (Scheme 1 and Scheme 2). The second step of the reaction is a biphasic one.

For both peroxyesters *tert*-butylhydroperoxide (R^1^—*tert*-butyl group) was used in the first reaction step to form the corresponding potassium salt. In the second reaction step pivaloyl chloride (R^2^—*tert*-butyl group) was used to produce TBPP and 2-ethylhexanoyl chloride (R^2^—CH_3_-CH_2_-CH_2_-CH_2_-CH(C_2_H_5_) group) to produce TBPEH.

Both reaction steps are exothermic. The total heat of reaction of step one is −23 kJ·mol^−1^ and corresponds to an adiabatic temperature rise of 25 K. The adiabatic temperature rise is a safety characteristic and describes the absolute value of temperature, by which the synthesis temperature is increased in case of failure. Presupposed is an adiabatic system (a worst case scenario), where all reaction heat is accumulated in the reaction mass. The conversion of the intermediate with the acid chloride, namely pivaloyl chloride (PivCl) to form TBPP and 2-ethylhexanoylchloride (EHC) to form TBPEH, is the more exothermic step with a heat of reaction of about −127 kJ·mol^−1^. This value corresponds to an adiabatic temperature increase of 74 K [25]. The organic phase of the reaction product decomposes with a high energy release. Measurements of thermal stability by Differential Scanning Calorimetry (heating rate 5 K·min^−1^, sample mass between 3 mg and 7 mg, high pressure sealed crucibles made of stainless steel) show decomposition energies of −1430 J·g^−1^ (TBPP, 98 *w*/*w*) and −1120 J·g^−1^ (TBPEH, 92 *w*/*w*), respectively [29].

Considering the fact of a biphasic reaction system in the second step, mixing of components plays an important role in process control. On the one hand, efficient mixing improves the contact of reactants and therefore a faster conversion, and intermediate accumulation is minimized, and on the other hand e.g., in case of failure, when decomposition starts, a good emulsification of the organic phase secures heat distribution also in the aqueous phase, which acts as heat sink due to its higher heat capacity. Therefore, all microreactor systems that were applied for the peroxyester synthesis used several mixing routes. Two reactors improve mixing in the second reaction step due to their specific channel structure; another reactor was stressed with ultrasonication.

#### 2.1.1. Orifice Microreactor

**Figure 1:**
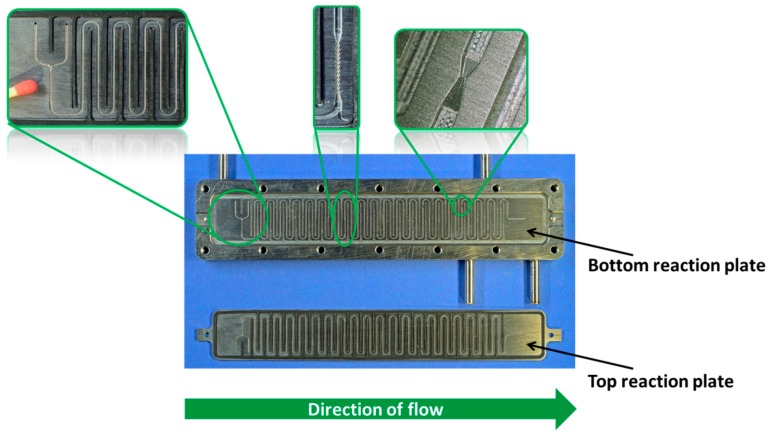
Orifice microreactor, top left zoom: 300 µm caterpillar micromixer for the mixing of the hydroperoxide with the potassium hydroxide, central zoom: 300 µm caterpillar micromixer for acid halide dosage, top right zoom: one of the five Venturi-like orifices.

The Orifice Microreactor (OMR), as one concept to synthesize organic peresters in continuous mode, was developed on the basis of a conceptual design set-up using baffle type orifices as re-emulsification units [22]. This concept was later modified and realized in the final OMR prototype, (Figure 1). It was developed and manufactured by IMM (now Fraunhofer ICT-IMM, Mainz, Germany) within the framework of a PhD thesis and supported by the Deutsche Bundesstiftung Umwelt (DBU file number 20007/894). The reaction plates were manufactured out of titanium grade 1 and purchased from HWN Titan (Mönchengladbach, Germany). The housing of the OMR was manufactured out of stainless steel (1.4435). Both seals were made of Viton. The connecting tubes for the utility fluid were V2A as well as the screwing and the guiding pins.

The OMR was tested in terms of synthesis performance, droplet disruption, residence time distribution, and heat removal, a detailed discussion is reported in references [23,24]. Its development was done under the consideration of the following boundary conditions:

- A small and compact design which should be scalable to higher throughputs.
- Combination of both reaction steps into one microreactor.
- The heat exchanger must be located below the reaction plate to enable the measurement of the heat profile along the top of the reaction plate which is covering the reaction channel.
- The reaction channel must be accessible for cleaning.
- For re-emulsification five orifices are used.
- Two caterpillar micromixers are used for generating a homogeneous mixture (first step) and for generating a pre-emulsion (second step).

Because the reaction channel is located half on one side of the reaction plate and the other half on the other side a high level of accuracy is needed to assure that the reaction channel and the orifice overlap when both plates are brought together. The positioning of the reaction plates is done by guiding pins located at both short sides of the OMR. The reaction plate of the OMR has the following dimensions (see Table 1).

**Table 1:** Channel lengths, surface areas and channel volumes realized in the orifice microreactor, calculated for a quadratic channel.

Section		L/m	A/m^2^	V/m^3^
KTBP-Formation	Mixer 1–Mixer 2	0.38	1.1 × 10^−3^	1.9 × 10^−7^
TBPP-Formation	Mixer 2–Orifice 1	0.15	4.2 × 10^−4^	7.5 × 10^−8^
	Orifice 1–Orifice 2	0.20	5.8 × 10^−4^	1.0 × 10^−7^
	Orifice 2–Orifice 3	0.16	4.6 × 10^−4^	8.2 × 10^−8^
	Orifice 3–Orifice 4	0.09	2.5 × 10^−4^	4.4 × 10^−8^
	Orifice 4–Orifice 5	0.06	1.7× 10^−4^	3.1 × 10^−8^
	Orifice 5–Outlet	0.03	9.6 × 10^−5^	1.7 × 10^−8^
	**Sum**	**1.08**	**3.1** × **10^−3^**	**5.4** × **10^−7^**

**Figure 2:**
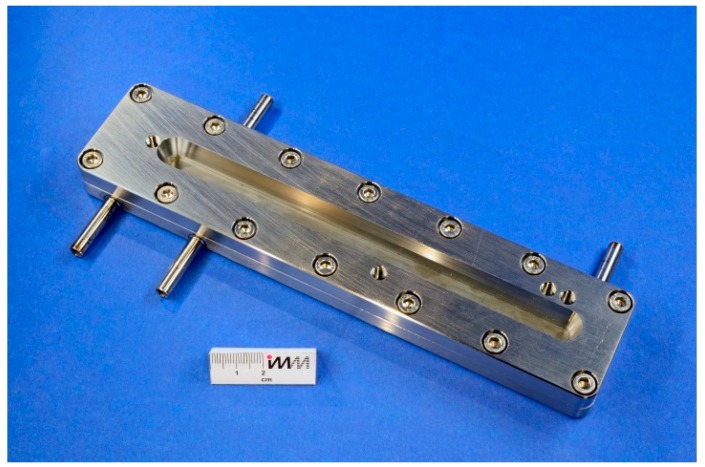
Mounted orifice microreactor, view on the inspection window for infrared measurements.

Both reaction sections in combination result in a channel length of 1.08 m, manufactured on a titanium reaction plate with a total length of 22.7 cm and a width of 3.16 cm. The OMR was tested under reactive conditions using two different flow rates, one of 10.5 mL·min^−1^ resulting in a residence time of 2.1 s (second step) and one of 18.5 mL·min^−1^ resulting in a residence time of 1.2 s (second step). The temperature profile on top of the reaction plate was measured via an inspection window (see Figure 2) by using an infrared camera.

**Figure 3:**
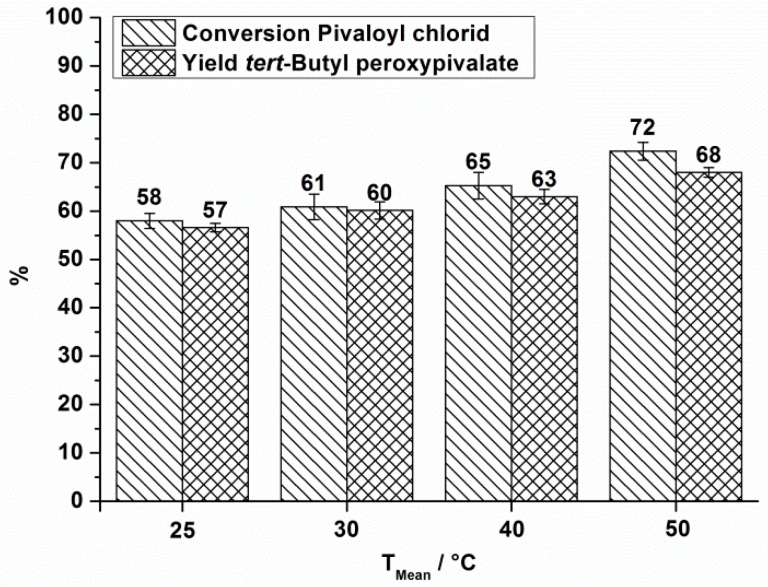
OMR, conversion and yield of TBPP synthesis, 2nd reaction step at a total flow rate of 10.5 mL·min^−1^ depending on the reaction temperature.

The obtained conversion and yield at different reaction temperatures is shown in Figure 3 for a total flow rate of 10.5 mL·min^−1^.

**Figure 4:**
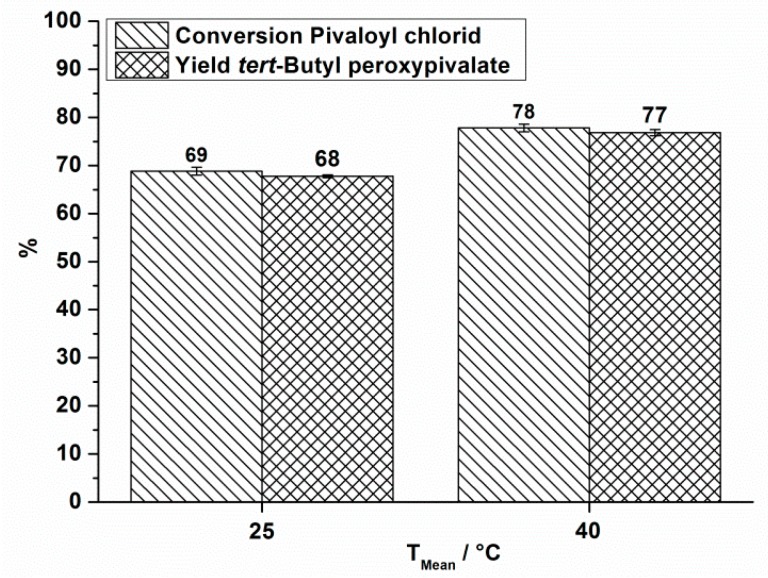
OMR, conversion and yield of TBPP synthesis, 2nd reaction step at a total flow rate of 18.5 mL·min^−1^ depending on the reaction temperature.

The same measurements were done at a total flow rate of 18.5 mL·min^−1^. For that case only reaction temperatures of 25 °C and 40 °C were investigated (Figure 4).

In all cases, a positive influence of increased reaction temperature on the process performance is seen. Processing at still higher reaction temperatures does not have a positive effect on the process performance. In processing without cooling a mean temperature of 81 °C is observed which is accompanied by strong gas formation. A decomposition of the organic phase, consisting mainly of TBPP is observed by Differential Scanning Calorimetry at temperatures above 40 °C (heating rate 2.5 K·min^−1^) [29]. Therefore it is supposed that decomposition occurs under the abovementioned conditions.

Because the second reaction is a biphasic one and the acid halide can accumulate within the organic phase it is evident that the quality of emulsion directly influences the process performance. One concept to compare different emulsifying devices with one another and to distinguish them from one another in terms of their efficiency on droplet disruption is the concept of energy density [30,31]. The following equations give the relationship between the energy density E_v_ (J·m^−3^), the power input P (J·s^−1^), the flow rate Q (m^3^·s^−1^) and the pressure drop Δp (Pa).

(1) $$E_{v}=\frac{P}{\dot{Q}}$$

(2) $$P=\;\Delta p\dot{Q}$$

(3) $$E_{v}=\Delta p$$

According to this theory the energy density at a flow rate of 10.5 mL·min^−1^ (Table 2) as well as for a total flow rate of 18.5 mL·min^−1^ (Table 3) is calculated for the varied reaction temperatures and the corresponding pressure drops.

**Table 2:** Energy density at a total flow rate of 10.5 mL·min^−1^ by using the OMR for TBPP synthesis at different reaction temperatures.

Entry	T_rct._/°C	Q_ges_/mL·min^−1^	Δp/Pa	E_v_/kJ·m^−3^	Conversion %		Yield %	τ_sect. 2_/s	τ_ges_/s
					TBHP	PivCl	TBPP		
1	25	10.5	180,000	180	54	58	57	2.1	3.5
2	30	10.5	150,000	150	56	61	60	2.1	3.5
3	40	10.5	140,000	140	59	65	63	2.1	3.5
4	50	10.5	120,000	120	66	72	68	2.1	3.5

**Table 3:** Energy density at a total flow rate of 18.5 mL·min^−1^ by using the OMR for TBPP synthesis at different reaction temperatures.

Entry	T_rct._/°C	Q_ges_/mL·min^−1^	Δp/Pa	E_v_/kJ·m^−3^	Conversion %		Yield %	τ_sect. 2_/s	τ_ges_/s
					TBHP	PivCl	TBPP		
1	25	18.5	380,000	380	71	69	68	1.2	2
2	40	18.5	300,000	300	79	78	77	1.2	2

Looking at the reaction temperature of 25 °C and that of 40 °C, at which the two flow rates were applied, an increase in the yield of TBPP is observed by increasing the total flow rate, even though the residence time shortens by a factor of 0.57. This positive influence can be explained by an enhanced mixing efficiency indicated by an increase in energy density from 180 kJ·m^−3^ to 380 kJ·m^−3^ for the case at 25 °C and from 140 kJ·m^−3^ to 300 kJ·m^−3^ at a reaction temperature of 40 °C. Also temperature increase causes higher yield and conversion, even if energy density decreases due to a change in viscosity. These measurements indicate that the considered perester synthesis depends on the one hand on the reaction temperature and on the other hand strongly on the energy input. The last can be set in relation to the mixing performance of the reactor.

The attempt to carry out the TBPEH synthesis with the OMR at BAM was restricted to a total flow rate of 5.25 mL·min^−1^ due to technical reasons. At 50 °C a TBPEH yield of 28% was obtained, which is remarkable low. Experiments with varying flow rates for TBPP production at Fraunhofer ICT-IMM showed that not until 10 mL·min^−1^ a good emulsification was gained. As a result, only then a distinct increase in conversion and yield was found. Taking into account the lower reactivity of EHC compared to that of PivCl, this characteristic can be expected for TBPEH production too. Probably the reactor (number and distance of orifices) and the reaction parameters (flow rate and temperature) have to be adapted to the reaction rate of EHC to end up at an optimized process.

#### 2.1.2. Split and Recombine Microreactor

The specific attribute of the Split and Recombine (SAR) reactor is the structure of its reaction channel. This structure splits fluid streams and combines them in an alternating manner. This is repeated along the whole reaction channel, hence, a permanent droplet break-up occurs. As a result, the contact surface of the liquid/liquid phases is continuously renewed and the emulsification is enhanced. A detailed description of the SAR reactor is given by Bošković [32]. The channel dimensions are 600 µm in width, 300 µm in height and 1.64 m in length. With the SAR reactor, only the second reaction step was carried out in continuous mode. Information about the production of the intermediate is specified in Section 3.3.

**Figure 5:**
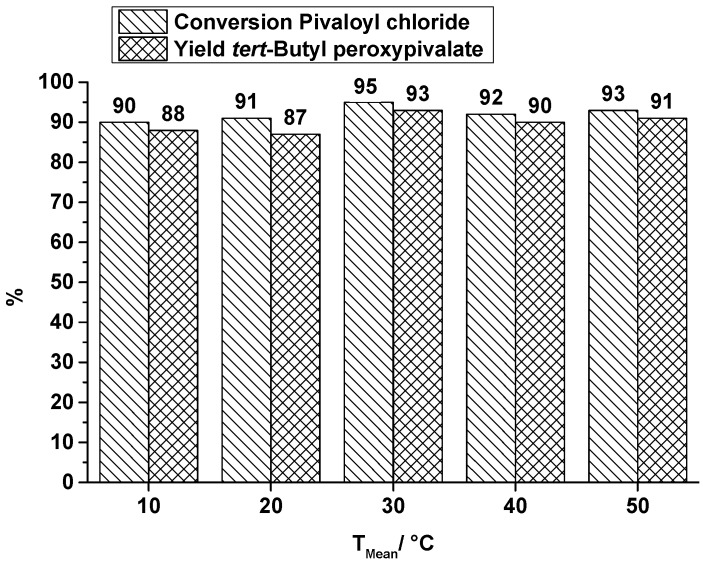
SAR reactor, conversion and yield of TBPP synthesis, 2nd reaction step at a total flow rate of 0.3 mL·min^−1^ depending on the reaction temperature.

**Figure 6:**
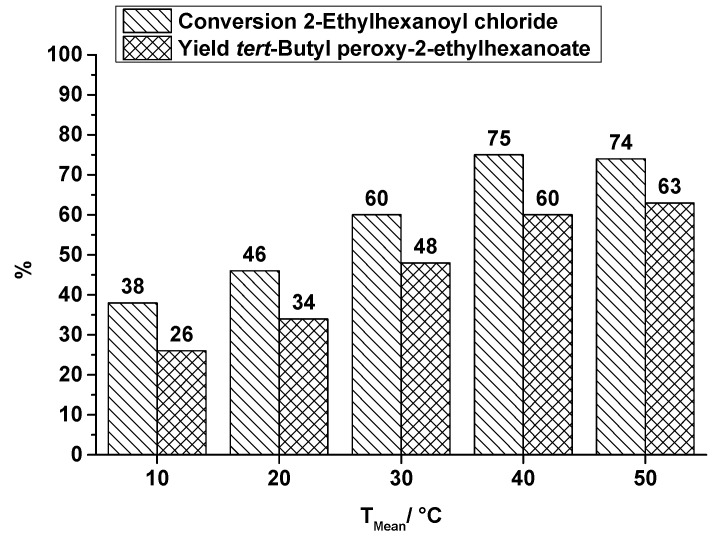
SAR reactor, conversion and yield of TBPEH synthesis, 2nd reaction step at a total flow rate of 0.3 mL·min^−1^ depending on the reaction temperature.

The conversion of the carboxylic acid chloride and the yield of the produced peroxyester at several synthesis temperatures at a total flow rate of 0.3 mL·min^−1^ are shown in Figure 5 (TBPP) and in Figure 6 (TBPEH). For the TBPP production, the conversion of PivCl as well as the yield of the peroxyester is high. Temperature influence is slightly noticeable, at least between 10 °C, 20 °C and 30 °C, whereas the higher temperature causes a better conversion to the desired product. Further temperature increase does not have a positive influence. Absolute values are higher than the corresponding values of the OMR, whereby e.g., flow rates are really different (OMR lowest flow rate 10.5 mL·min^−1^).

Comparing the production of the two peroxyesters TBPP and TBPEH under the same conditions in the SAR reactor, the lower conversion of the long-chain acid chloride, 2-ethylhexanoyl chloride (EHC), and the corresponding lower yield of TBPEH is obvious. It is explained by a difference in the reactivity of the used acid halides. Pivaloyl chloride is more reactive than 2-ethylhexanoyl chloride. An increase in reaction temperature increases the yield of TBPEH too, e.g., at a reaction temperature of 10 °C a yield of 26% is obtained, whereas at a reaction temperature of 50 °C the yield of 63% is significantly higher. A further increase in reaction temperature does not necessarily enhance the productivity. As known from DSC measurements, decomposition of TBPEH becomes detectable at about 60 °C [29] and counteracts a higher yield.

**Figure 7:**
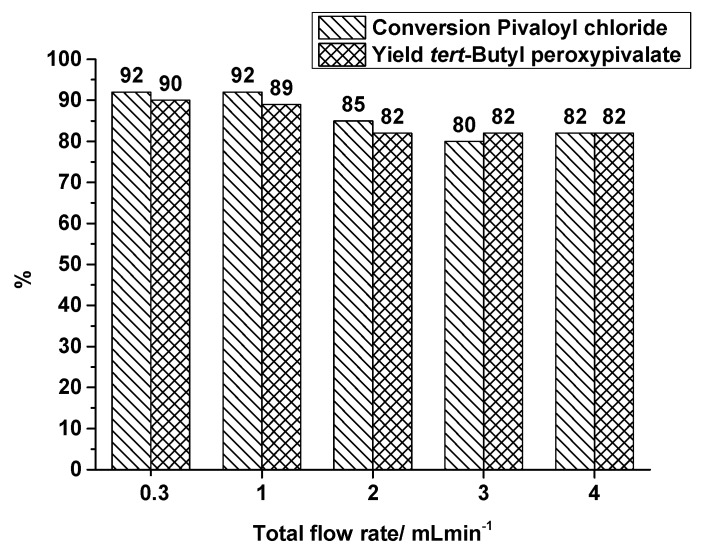
SAR reactor, conversion and yield of TBPP synthesis, 2nd reaction step at a reaction temperature of 40 °C in dependence on total flow rate.

As mentioned before, the flow rates in the OMR were quite different compared to the SAR reactor. Therefore, additional runs in the SAR reactor producing TBPP were carried out with higher total flow rates than 0.3 mL·min^−1^, covering 1, 2, 3 and 4 mL·min^−1^ at a constant synthesis temperature of 40 °C. Figure 7 shows a decrease of conversion and yield at flow rates higher than 1 mL·min^−1^. By increasing the total flow rate the residence time decreases, but a better mixing can be assumed. Looking at the flow rates of 0.3 mL·min^−1^ and 3 mL·min^−1^, the residence time decreases by a factor of 10, from 64 s to 6.4 s, but TBPP production is still at a high level. By changing the flow rate from 0.3 mL·min^−1^ to 3 mL·min^−1^, the yield of TBPP decreases from 90% to 82%. In spite of this reduction the space-time yield increases from nearly 17,000 kg·m^−3^·h^−1^ to 152,000 kg·m^−3^·h^−1^.

In the same way as described in Section 2.1.1, for the OMR measurements the energy density can be calculated for the TBPP synthesis using the SAR reactor at different flow rates (Table 4), while synthesis temperature is kept constant.

**Table 4:** Energy density at varying total flow rates by using the SAR reactor for TBPP synthesis at a reaction temperature of 40 °C.

Entry	T_rct._/°C	Q_ges_/mL·min^−1^	Δp/Pa	E_v_/kJ·m^−3^	Conversion %	Yield %	τ_ges_/s
					PivCl	TBPP	
1	40	1	124,226	124	92	89	19.2
2	40	2	146,917	147	85	82	9.6
3	40	3	174,491	174	80	82	6.4
4	40	4	205,021	205	82	82	4.8

With increasing flow rate the energy density in the SAR reactor experiments is increasing as well, but in contrast to the OMR experiments these rises do not cause an increase in conversion and yield. On the contrary, the yield decreases at least when accelerating the total flow rate from 1 mL·min^−1^ to 2 mL·min^−1^. Further increase shows no effect, even though residence time decreases further. That may be an indication that at a certain level of conversion the provided residence time gets more important compared to an increase in energy density to enhance the mixing performance. A comparison of the OMR and SAR reactor is difficult because of complex interaction of flow rate and residence time, pressure drop and energy density and the specific mixing structure. Hence, the specification of the energy density alone is not sufficient for a differentiation of different reactor types processing reactive species.

#### 2.1.3. Capillary Tube Reactor in Combination with Ultrasonication

**Figure 8:**
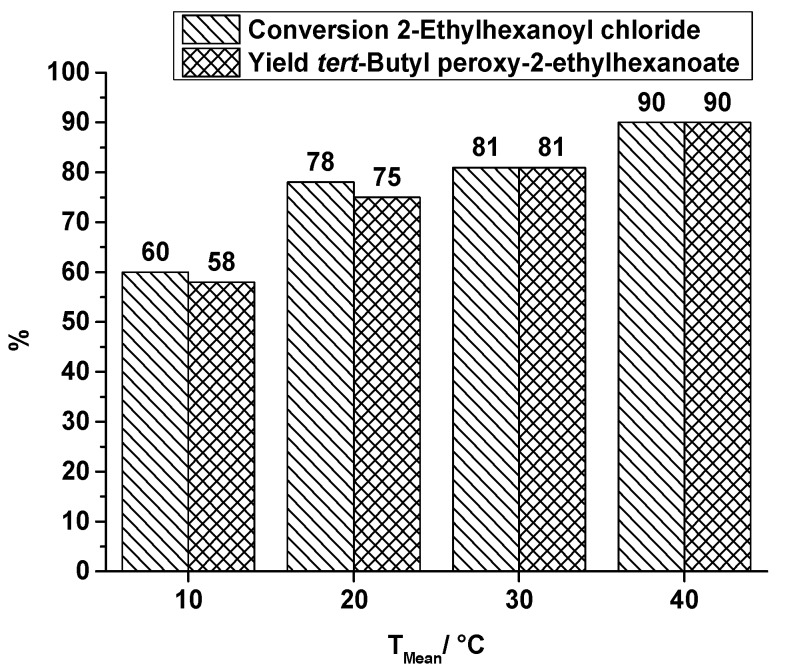
Capillary tube reactor with ultrasonication, conversion and yield of TBPEH synthesis, 2nd reaction step at a total flow rate of 0.3 mL·min^−1^ depending on the reaction temperature.

The application of ultrasound (US) to break-up the biphasic reaction system of the second reaction step is a completely different approach that can be used to enhance mixing performance. Former runs without this specific kind of reactant stress showed a low conversion of acid chloride and a low yield of the corresponding peroxyester, e.g., at a flow rate of 0.3 mL·min^−1^ and a synthesis temperature of 30 °C the conversion of EHC is 61% and the yield of TBPEH is 57% [25]. That conversion and yield is improved by ultrasonication is shown in Figure 8, covering a temperature range from 10 °C to 40 °C at a total flow rate of 0.3 mL·min^−1^. In comparison to the results obtained without US, a significant increase in yield up to 90% of TBPEH is obtained.

A temperature influence on conversion and yield is visible, as already observed in the runs of TBPEH production in the SAR reactor. Noticeable is the low numerical difference between conversion and yield, which corresponds to a high selectivity of peroxyester formation. With respect to the length of the capillary tube, which was immersed in the ultrasonic bath (0.58 m of total tube length), the best space-time-yield obtained is 12,500 kg·m^−3^·h^−1^.

A drawback of ultrasonication of liquids is the generation of bubbles, which can collapse producing a local pressure and temperature spot. Rivas *et al.* [33] reported the generation of microbubbles and light emission in a microstructured reactor, whereas only water, aqueous luminal or propanol was used. In case of liquid organic peroxides the flow through of bubbles (cavitated systems) can be a potential risk for a reinforced decomposition behavior. Such systems can facilitate the transition from a thermal decomposition via deflagration into a detonation, which is a more energy-rich process with severe consequences compared to a thermal decomposition. Also induced detonations can be propagated better in cavitated liquid organic peroxides [34]. The specific compounds, technical pure TBPP and TBPEH, which are comparable to the organic phase of the reaction mixture, propagate a detonation if the liquid is tested in the cavitated state following UN-Test Series 1, Type 1(a), Appendix 3 [35]. Such tests are carried out to classify substances or mixtures in connection with a safe transport, storage and handling according to international appointed test methods. The mentioned test run in tubes with an inner diameter of 50 mm, meaning an enlargement in diameter by a factor of 50 compared to the used capillary tube reactor. At the moment it is not known if the effect of cavitation on the decomposition behavior appears in small dimensioned tubes as well, in particular for the named compounds. Nevertheless, the above given results show the possibility of using US for this type of reaction, at least when done on a bench scale.

## 3. Experimental Section

Because different laboratories were involved in this research, different chemical suppliers and analytical systems were used. Thus, the following sections distinguish between measurements at Fraunhofer ICT-IMM (OMR/TBPP) and BAM (Berlin, Germany, SAR, capillary tube/TBPP and TBPEH).

### 3.1. Test Procedure and Set-Up Using the Orifice Microreactor

Using the OMR at Fraunhofer ICT-IMM both reaction steps are performed in consecutive manner. An aqueous solution of 22.7 *w*/*w* KOH and an aqueous solution of 68 *w*/*w* TBHP are feed with HPLC-pumps (K-501 with a 10 mL stainless steel pump head, Knauer, Berlin, Germany) into the OMR and contacted in a 300 µm caterpillar micromixer (Fraunhofer ICT-IMM) with twelve splitting and recombination sections. The feed rates are adjusted in such a way that the KOH is in a slight excess of 1.12 eq. based on the amount of TBHP. The reaction mixture is then fed into the second 300 µm caterpillar micromixer with twelve splitting and recombination sections and contacted with the PivCl. The substoichiometric amount of PivCl (0.85 eq.) is fed via a Knauer Smartline 1000 pump with a 10 mL titanium pump head. The reaction temperature is controlled by using a temperature controlled water bath connected to the OMR heat exchanger. The total flow rates used are 10.5 mL·min^−1^ and 18.5 mL·min^−1^, leading to residence times for the second step of the reaction of 2.1 s and 1.2 s, respectively.

### 3.2. Test Procedure and Set-Up Using the Split and Recombine Reactor and a Capillary Tube Reactor Combined with Ultrasonication

With both reactor types only the second reaction step was carried out continuously. The intermediate, the aqueous *tert*-butyl potassium peroxide solution (TBKP), was produced in semi-batch mode.

As capillary tube a simple PFA tube with an inner diameter of 1 mm (outer diameter 1/16 inch) and a total length of 1.25 m was used. It was immersed in an ultrasonic bath (RK 156 BH, 35 Hz, Bandelin Electronic, Berlin, Germany), the effective tube length in the bath was 0.58 m. The SAR, made of glass, was purchased from Mikroglas Chemtech GmbH (Mainz, Germany).

In case of the capillary tube reactor, the reactants were first mixed in a y-shaped mixer and then transferred into the PFA tubing where ultrasonication occurred. The SAR reactor, equipped with two inlets, was supplied directly with the reactants. Syringe pumps from Little Things Factory (Elsoff, Germany) were used for fluid supply. Individual flow rates of intermediate and acid chloride were adjusted for TBPEH synthesis in a ratio of 1 mol TBKP to 1 mol EHC. For TBPP synthesis molar ratio of TBKP to PivCl was 1.15:1. The total flow rate varied between 0.3 mL·min^−1^ and 4 mL·min^−1^ for the SAR reactor experiments, while only a flow rate of 0.3 mL·min^−1^ was used for the experiments in the capillary tube reactor with US.

Prior to the reactant contact within the microreactor, both fluid streams were pre-heated/pre-cooled in a bath, in which the reactor was immersed too. The bath temperature was regulated by a thermostat. The temperature varied between 10 °C and 50 °C in 10-K steps when the SAR reactor was used and between 10 °C and 40 °C when the capillary tube was used as reactor. After leaving the SAR reactor or in case of the capillary tube, after leaving the ultrasonic bath, the reaction mixture was cooled down with a tube in tube heat exchanger to stop or at least to reduce further conversion on the way to the separation funnel. The last was already filled with approximately 100 mL deionized water to quench any still ongoing reaction. The organic and aqueous phases were separated.

### 3.3. Preparation of the Intermediate tert-Butyl Potassium Peroxide (TBKP) in Semi-Batch Mode

The intermediate was synthesized in a stirred tank reactor. Aqueous KOH solution (22.7 *w*/*w*) was provided in the reactor, tempered to 25 °C and converted with aqueous TBHP solution (68 *w*/*w* to 69 *w*/*w*) under nearly isothermic conditions. Concentration of TBKP in the intermediate solution was analyzed by iodometric redox titration. For the SAR reactor experiments the TBKP concentration was 31.1 *w*/*w* and for the experiments in the capillary tube reactor 31.8 *w*/*w*.

### 3.4. Chemicals

KOH (puriss. p.a., Reag. Ph. >85%), TBHP (70 *w*/*w* in water) and PivCl (99% GC) were purchased from Sigma Aldrich (Munich, Germany). MeCN (Rotisolv^®^ gradient grade) was purchased from Carl Roth GmbH & Co. (Karlsruhe, Germany) and TBPP (70 *w*/*w*–80 *w*/*w* in isododecane as analytical reference substance) was obtained from Pergan (Bocholt, Germany). KOH (puriss. p.a., Reag. Ph.) and acetonitrile (Rotisolv HPLC 99.9%) were purchased from Carl Roth GmbH & Co. HCl (1 N, Titrisol) was purchased from Merck KGaA (Darmstadt, Germany).

For the measurements at BAM several aqueous TBHP solutions of a peroxide producer with a concentration of about 70 *w*/*w* were used. The exact concentration was analysed by iodometric redoxtitration before the TBHP was used as reactant in the first reaction step. Aqueous KOH solution (22.7 *w*/*w*) was prepared from solid KOH pellets, purchased from Merck KGaA. Its concentration was verified by acid-base titration. PivCl purum (≥98% GC) from Acros Organics (Geel, Belgium) and 2-ethylhexanoyl chloride (99% GC) from Merck KGaA were used respectively in the second step.

### 3.5. Analytical Part

Reaction mixtures (organic and aqueous phase), produced with the OMR at Fraunhofer ICT-IMM were analysed via HPLC. At BAM, only the organic phase of the reaction mixture was analyzed via HPLC. Peroxide concentration in TBHP solution and intermediate (TBKP) was analysed by iodometric redoxtitration.

Samples from the OMR experiments are pre-treated as follows: 1 g–1.5 g of reaction mixture is diluted with 20 mL of a 7:3 mixture of MeCN and water (adjusted to pH 2.5 with 1 N aqueous HCl) and then injected into the HPLC. The eluent consists of MeCN and water (pH 2.5 adjusted with 1 N aqueous HCl). The flow rate of 1 mL·min^−1^ results in a starting pressure of 15.2 MPa using a fluid gradient with following operation conditions: Pump B: *t* = 2 min (MeCN) 25%; Pump B: *t* = 5 min (MeCN) 70%; Pump B: *t* = 8 min (MeCN) 70%; Pump B: *t* = 10 min (MeCN) 25%; Controller stop: *t* = 18 min; oven temperature: 25 °C. The reaction components are detected using a PDA detector at a wavelength of 230 nm; Cell temperature 40 °C; slit width 1.2 nm; Lamp: D2. Shimadzu Deutschland GmbH (Duisburg, Germany) LC System: System Controller: CBM-20A, Pump A and B: LC-20AD, Auto sampler: SIL-20A HT, Column oven: CTO-20AC, PDA: SPD-M20A. The used reversed phase HPLC column is a 250 × 4.0 mm Nucleosil 120C 18 5 µm (MZ-Analysentechnik, Mainz, Germany, Art. No. 250.4 0.3135.N, Ser. No. 15130818).

At BAM, a sample of the organic phase of the reaction mixtures (15 µL) is taken and poured in a 10 mL graduated flask, diluted with 0.5 mL methyl alcohol for a better dissolution and finally filled up with the HPLC eluent. The eluent consists of a methyl alcohol and water mixture 55:45 *w*/*w* for TBPP samples and of 65:35 *w*/*w* for TBPEH samples. The eluent is buffered to pH 2.6 with a H_3_PO_4_ solution (0.6 mL H_3_PO_4_/L eluent). During the entire analytical run on the Waters HPLC instrument (Pump: 510, Auto sampler: 717plus, Column oven: Jetstream plus, Absorbance detecector: 486, Waters Cooperation, Milford, MA, USA, the eluent composition is kept constant, no gradient is applied. Injected sample volume is 10 µL. As separation column a reversed phase HPLC column (Kinetix 2.6u XB-C18 100Å, 00C-4496-E0, Phenomenex Inc., Torrance, CA, USA) with the dimensions 4.6 mm Id, 50 mm length, 2.6 µm particle size, 100 Å pore size is applied. Oven temperature is kept constant at 30 °C. The UV detector is set to 207 nm in the first 2.1 min and afterwards to 220 nm. With a flow rate of 1 mL·min^−1^ retention times of analyzed components in the TBPP samples are 1.24 min for TBKP, 1.6 min for pivalic acid, 2.45 min for PivCl and 5.30 min for TBPP. For TBPEH samples retention times are as follows: 1.1 min for TBKP, 2.69 min for 2-ethylhexanoic acid, 5.06 min for EHC and 9.67 min for TBPEH. Iodometric redoxtitration of TBHP or TBKP is carried out with a Methrom AG (Zofingen, Switzerland) system (736 GP Titrino, Pt ring electrode). The peroxide reacts with a SO_2_ solution, added in molar excess. Back tritration of the non-reacted SO_2_ with iodine allows the calculation of the peroxide concentration. The titration beaker filled with 20 mL toluene and 40 mL methyl alcohol before a 0.1 N SO_2_ solution (prepared from Karl-Fischer solution A, 990 mL methyl alcohol and 90 mL pyridine) is added. The weighted sample is filled into the beaker. After a reaction time of a few minutes peroxide is converted and the remaining SO_2_ is titrated with 0.1 N iodine solution. Conversion is followed potentiometrically (automatic equivalence point finding monotonic titration, MET). In the same manner a blind sample is analyzed.

## 4. Conclusions

With the application of the OMR, the SAR reactor and the capillary tube reactor in combination with US, three different mixing strategies were tested. In the OMR and in the SAR reactor, emulsification is achieved due to the static mixing elements that are implemented into the reaction channel. For the capillary tube reactor, mixing is induced by the stress that is applied by US. For the presented reaction, the synthesis of peroxyester from an aqueous solution of a precursor and an acid halide, the emulsification of the components plays an important role. An increase in mixing performance helps to shorten the reaction time down to seconds.

It is known from conventional used orifices that an increase in flow rate leads to a higher turbulence in the fluid behind the orifice. This fact was observed also in the peroxyester production with the OMR. As a consequence, a high yield could be achieved at high flow rates and very short residence times. Under the best process conditions, a space-time-yield of 414,000 kg·m^−3^·h^−1^ is obtained. Such high values make microreaction technology very attractive for industrial applications.

In the SAR reactor, the increase in total flow rate led only up to a certain value to an increase in conversion and yield. A further increase of flow rate caused a contrary trend, as conversion and yield decreased due to shorter residence time.

The influence of reaction temperature on the reactor performance is clearly visible in all three discussed systems. This influence cannot directly be correlated to a special design criteria of any one of the investigated microreactors, because they differ in inner volume and mixing structure. Therefore, we tried to use the concept of energy density as one characteristic variable to compare the microreactor performances with each other. For the OMR and the SAR reactor the energy density, which is proportional to the pressure drop, was calculated for the TBPP synthesis. For certain synthesis parameters at least similar energy density values were obtained in both reactor types. Consideration of conversion and yield showed that the energy density as one single parameter is not a suitable characteristic variable to compare different microreactors. Due to the reactivity of the chemical system the influence of energy density on the reactor performance could not be fully decoupled from e.g., kinetic parameters of the investigated reactions. To overcome this effect, it is necessary to limit this multiparameter problem to e.g., a one parameter problem. It is therefore necessary to keep parameters like residence time, energy density, and reaction temperature constant for each type of microreactor. Furthermore, the produced interfacial area in case of the biphasic system, takes influence on the reaction performance. Thus, it has to be checked if all microreactors produce the same interfacial area by the same energy input. If that is the case, the influence of mass transfer should be similar for all types of microreactors. For the capillary tube reactor the produced interfacial area should be set in correlation to the energy input by ultrasound.

The capillary tube reactor with ultrasonication showed the best results concerning the conversion of EHC and the yield of TBPEH. Noticeable is the high selectivity for the TBPEH synthesis with this reactor type. A drawback of this set-up is the potential risk of severe decomposition of the peroxyester, initiated by the collapse of bubbles, which are produced in the liquid reaction mixture by ultrasonication. Such an occurrence was not observed during the test runs, but cannot be excluded.

In general, several microstructured reactors are suitable for the production of TBPP and TBPEH, and high space-time yields can be achieved. Production of high quantities of peresters requires reasonable scale-up or numbering up of the microreactor. It is a challenge for process monitoring and control technology and also a question of quantity of reactor material. In the case of peroxide synthesis, used often in smaller amounts for the initiation of a polymerization process, a production on demand could be a promising solution.
